# Mit künstlicher Intelligenz schneller zur Diagnose seltener Erkrankungen – ein Gebot der Ethik, Ökonomie und Lebensqualität

**DOI:** 10.1007/s00108-023-01599-7

**Published:** 2023-10-20

**Authors:** Lukas Völkel, Annette D. Wagner

**Affiliations:** 1 Medizinische Hochschule Hannover, Carl-Neuberg-Str. 1, 30625 Hannover, Deutschland; 2 Abteilung für Nieren- und Hochdruckerkrankungen, Ambulanz für seltene entzündliche Systemerkrankungen mit Nierenbeteiligung, Medizinische Hochschule Hannover, Carl-Neuberg-Str. 1, 30625 Hannover, Deutschland

**Keywords:** Künstliche Intelligenz/ethische Aspekte, Gesundheitskosten, Gesundheitsbezogene Lebensqualität, Datenschutz, Digitale Diagnoseunterstützungssysteme, Artificial intelligence/ethics, Healthcare costs, Quality of life, health-related, Data protection, Digital diagnostic support systems

## Abstract

**Hintergrund:**

Weltweit leiden etwa 300 Mio. Menschen an einer seltenen Erkrankung. Eine optimale Therapie setzt eine erfolgreiche Diagnose voraus. Diese dauert bei seltenen Erkrankungen besonders lange. Digitale Diagnoseunterstützungssysteme könnten zukünftig wichtige Helfer bei der Beschleunigung einer erfolgreichen Diagnose sein.

**Ziel der Arbeit:**

Die aktuellen Möglichkeiten digitaler Diagnoseunterstützungssysteme in der Diagnostik seltener Erkrankungen und noch zu klärende Fragestellungen sollen in Bezug auf die Parameter Ethik, Ökonomie und Lebensqualität dargelegt werden.

**Material und Methoden:**

Aktuelle Forschungsergebnisse des Autorenteams werden im Kontext aktueller Literatur zusammengetragen und diskutiert. Anhand eines Fallbeispiels wird das Potenzial digitaler Diagnoseunterstützungssysteme erläutert.

**Ergebnisse:**

Digitale Diagnoseunterstützungssysteme und Expert:innen zusammen können die erfolgreiche Diagnose bei Patient:innen mit seltener Erkrankung beschleunigen. Dies könnte positive Auswirkungen auf die Lebensqualität der Patient:innen haben und zu Einsparungspotenzial bei den direkten und indirekten Kosten im Gesundheitssystem führen.

**Schlussfolgerung:**

Die Gewährleistung von Datensicherheit, Rechtssicherheit und Funktionalität bei der Verwendung digitaler Diagnoseunterstützungssysteme ist von hoher Bedeutung, wenn Vertrauen bei Expert:innen und Patient:innen geschaffen werden soll. Eine stetige Weiterentwicklung der Systeme mittels künstlicher Intelligenz könnte zukünftig auch Patient:innen dazu befähigen, die Diagnosefindung aktiv zu unterstützen.

## Abstract (en)

**Background:**

Approximately 300 million people worldwide suffer from a rare disease. An optimal treatment requires a successful diagnosis. This takes a particularly long time, especially for rare diseases. Digital diagnosis support systems could be important aids in accelerating a successful diagnosis in the future.

**Objective:**

The current possibilities of digital diagnostic support systems in the diagnosis of rare diseases and questions that still need to be clarified are presented in relation to the parameters of ethics, economy and quality of life.

**Material and methods:**

Current research results of the authors were compiled and discussed in the context of the current literature. A case study is used to illustrate the potential of digital diagnostic support systems.

**Results:**

Digital diagnostic support systems and experts together can accelerate the successful diagnosis in patients with rare diseases. This could have a positive impact on patients’ quality of life and lead to potential savings in direct and indirect costs in the healthcare system.

**Conclusion:**

Ensuring data security, legal certainty and functionality in the use of digital diagnostic support systems is of great importance in order to create trust among experts and patients. Continuous further development of the systems by means of artificial intelligence (AI) could also enable patients to accelerate diagnosis in the future.

Schätzungen zufolge sind weltweit 8000 seltene Erkrankungen bekannt [10, 22]. In Deutschland leiden etwa 4 Mio. Menschen an einer seltenen Erkrankung, in Europa etwa 30 Mio. und weltweit etwa 300 Mio. [20]. Seltene Erkrankungen werden weltweit unterschiedlich definiert. In den USA gilt eine Erkrankung als selten, wenn landesweit nicht mehr als 200.000 Einwohner:innen betroffen sind [7]. In Europa dagegen spricht man von einer seltenen Erkrankung, wenn weniger als eines von 2000 Individuen betroffen ist [1]. Seltene Erkrankungen stellen eine erhebliche gesellschaftliche Belastung dar und haben teils verheerende Auswirkungen auf Patient:innen, ihre Familien und das Gesundheitssystem [5].

Ein Bericht der Europäischen Konferenz über seltene Erkrankungen aus dem Jahr 2005 analysierte die Lebenserwartung der Patient:innen bei 323 seltenen Erkrankungen und stellte fest, dass 25,7 % der seltenen Erkrankungen potenziell vor dem fünften Lebensjahr tödlich verliefen und weitere 36,8 % zu einer reduzierten Lebenserwartung führten, während nur etwa ein Drittel (37,5 %) mit einer normalen Lebenserwartung verbunden war [3]. Eine italienische Studie zeigte, dass die Todesfälle in einer Population mit seltenen Erkrankungen 4,2 % aller verlorenen Lebensjahre der Allgemeinbevölkerung ausmachten. Das sind fast 4‑mal so viele verlorene Lebensjahre wie durch Infektionskrankheiten und fast doppelt so viele wie durch Diabetes [14]. Zudem sind über 70 % der seltenen Erkrankungen genetisch bedingt [20].

Es ist sehr schwierig, seltene Erkrankungen schnell zu diagnostizieren

Es ist für Ärzt:innen sehr schwierig, seltene Erkrankungen schnell zu diagnostizieren, da diese aufgrund ihrer Komplexität, Variabilität, unspezifischen Symptomatiken und des mangelnden Wissens über seltene Erkrankungen im Allgemeinen eine große Herausforderung darstellen [15, 23]. Ein Viertel aller Patient:innen mit seltenen Erkrankungen wartet zwischen 5 und 30 Jahren, bis eine richtige Diagnose gestellt wird [3]. Eine Survey-Studie aus den USA und Großbritannien konnte zeigen, dass ein:e Patient:in im Durchschnitt bei 8 Ärzt:innen, 4 Ambulanzen und 4 Fachärzt:innen vorstellig wird, bevor sie/er die richtige Diagnose erhält [16]. Auf dem Weg dorthin wird ein:e Patient:in zusätzlich mit 2 bis 3 Fehldiagnosen belastet.

In interdisziplinären Spezialzentren für seltene Erkrankungen arbeiten Expert:innen vieler verschiedener Fachrichtungen, die sich interdisziplinär austauschen können und über einen großen Wissens- und Erfahrungsschatz verfügen [2]. Diese Zentren weisen alle notwendigen Strukturen und Ressourcen auf, um die Patient:innen mit seltenen Erkrankungen optimal diagnostizieren und behandeln zu können. Allerdings bleibt trotz der Zunahme an diagnostischen Methoden und Tests eine frühzeitige Diagnosefindung herausfordernd.

Große Chancen werden im Einsatz von digitalen Diagnoseunterstützungssystemen gesehen

Große Chancen werden im Einsatz von digitalen Diagnoseunterstützungssystemen gesehen. Diese könnten zahlreiche positive Effekte haben:

- Schnellere Diagnosen
- Kompetentere Beratungen
- Schnellere Überweisungen an Spezialzentren
- Kürzere Behandlungspfade
- Eine bessere Einbindung in internationale Netzwerke

Die systematische Digitalisierung erfordert ein hohes Maß an Koordination aller Beteiligten. Einen Beitrag hierzu leistet etwa das Portal für seltene Erkrankungen und Orphan Drugs (https://www.orpha.net). Orphanet Deutschland, das als nationaler Partner des europäischen Referenzportals für seltene Erkrankungen unter anderem die Orphanet-Nomenklatur für Deutschland bereitstellt, wurde am 01.01.2021 an das Bundesinstitut für Arzneimittel und Medizinprodukte (BfArM) angegliedert.

Bereits seit einigen Jahren werden im Bereich der seltenen Erkrankungen Systeme zur Entscheidungsunterstützung angeboten und fortlaufend weiterentwickelt, von denen man sich eine deutlich schnellere und erfolgreiche Diagnosestellung verspricht. Verbreitet sind Systeme wie Isabel, Phenomizer und Orphanet. Im systematischen Einsatz von Diagnoseunterstützungssystemen sind die Zentren für seltene Erkrankungen den anderen Bereichen der Medizin deutlich voraus. Der Einsatz künstlicher Intelligenz (KI) soll Ärzt:innen bei ihren diagnostischen Überlegungen optimal unterstützen [4, 6, 18]. Die Funktionalitäten mancher Anwendungen sind auf das Erkennen spezifischer seltener Erkrankungen ausgelegt, während andere das Identifizieren von seltenen Erkrankungen im Allgemeinen fokussieren [4]. Die Ziele der diagnostischen Unterstützungssysteme sind vielfältig:

- Höhere Effektivität
- Geringere Kosten
- Weniger Zeitaufwand
- Höhere Genauigkeit
- Größere Verbreitung

In diesem Beitrag sollen die drei wesentlichen Faktoren – Lebensqualität, Ökonomie und Ethik – im Kontext des Einsatzes von KI zur schnelleren Diagnose seltener Erkrankungen betrachtet werden.

## Lebensqualität

Das Robert Koch-Institut (RKI) definiert gesundheitsbezogene Lebensqualität als ein „multidimensionales Konstrukt aus physischen, psychischen und sozialen Dimensionen“ [17]. Die Lebensqualität umfasst demnach individuelle und gesellschaftliche Lebensverhältnisse sowie subjektive und objektive Komponenten.

Der korrekte Einsatz von KI kann im klinischen Alltag den Weg zur Diagnose verkürzen, Lebensqualität steigern und Gesundheitskosten senken. Neue Technologien des maschinellen Lernens haben das Potenzial, die Erforschung, Diagnostik und Behandlung seltener Erkrankungen deutlich zu verbessern. Verschiedene Studien haben bereits unterschiedliche Ansätze gezeigt, um mithilfe von Algorithmen der KI schnell Muster und Zusammenhänge zu erkennen oder mithilfe prädiktiver Modellierungstechniken wie Deep Learning den weiteren Krankheitsverlauf vorhersagen zu können [11, 12, 19, 21].

Die korrekte Diagnosestellung kann die subjektive Lebensqualität Betroffener maßgeblich verbessern

In einer eigenen Arbeit konnten wir zeigen, dass das Expertensystem von Ada (Ada DX, Ada Health GmbH, Berlin, Deutschland) in 54 % der Fälle einen frühzeitigen korrekten Top-5-Diagnosevorschlag lieferte, in 33 % der Fälle sogar bereits im Rahmen der ersten dokumentierten Vorstellung bei einem Arzt bzw. einer Ärztin. Die Genauigkeit der Vorschläge von Ada DX zum Zeitpunkt der Diagnose lag bei 89 % [18]. Der Zeitpunkt der richtigen Diagnosestellung kann dabei als kritischer Punkt betrachtet werden. Zwar steht nicht für jede Erkrankung eine adäquate Therapie zur Verfügung, dennoch ist ein:e Patient:in meist erleichtert, die korrekte Diagnose zu erhalten. Dieses Wissen kann maßgeblich zur subjektiv empfundenen Lebensqualität der Betroffenen beitragen und die Patient:in kann genauer therapiert werden und den Umgang mit seiner/ihrer Erkrankung erlernen. Die Lebensqualität des Patienten/der Patientin kann sich auf physischer, psychischer wie auch sozialer Ebene verbessern, wenn die Diagnosefindung erfolgreich abgeschlossen wird.

## Ökonomie

**Figure 1:**
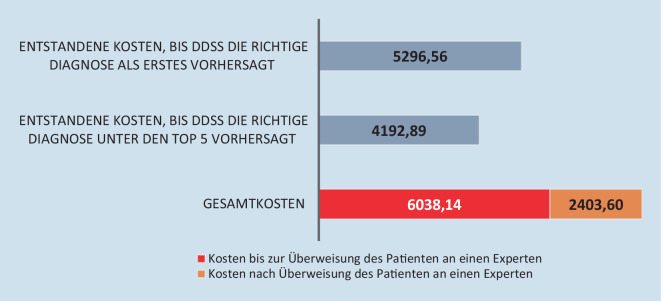

Neben der Lebensqualität der Patient:innen werden auch die Kosten für das Gesundheitssystem von der richtigen Diagnosestellung direkt beeinflusst. Ein klassischer Ansatz für die Diagnose einer seltenen Erkrankung umfasst Anamnese, körperliche Untersuchungen und Genanalysen, mit denen bestimmte Mutationen, die mit einer Erkrankung in Verbindung stehen, eruiert werden können. Zusätzlich werden bildgebende Untersuchungen wie Röntgenaufnahmen, Magnetresonanztomographie(MRT)-, Computertomographie(CT)- oder Positronenemissionstomographie(PET)-CT-Scans eingesetzt. Direkte Kosten entstehen für alle medizinischen Leistungen zur Behandlung von Erkrankungen. Umfangreiche Tests und bildgebende Untersuchungen werden bei seltenen Erkrankungen häufig unnötigerweise mehrfach wiederholt, wodurch höhere Kosten für das Gesundheitssystem entstehen. In einer eigenen Arbeit [24] konnten die direkten Kosten betrachtet werden, die für Patient:innen auf ihrer Odyssee bis zur richtigen Diagnose entstehen. In der Arbeit wurde gezeigt, dass Ada DX und auch die Überweisung an eine/einen Expert/Expertin erhebliche Einsparungen bei der Diagnose seltener Erkrankungen erzielen können (Abb. 1). Die Evaluierung der direkten Kosten in einem Kollektiv von Patient:innen mit seltenen Erkrankungen ergab, dass die Gesundheitskosten durch den Einsatz des Diagnoseunterstützungssystems Ada DX um bis zu 50 % geringer ausfielen. Ursächlich hierfür ist, dass unnötige wiederholte Analysen und auch zum Teil invasive Untersuchungen bei den Patient:innen vermieden werden konnten.

Patient:innen mit seltenen Erkrankungen leiden häufig unter Produktivitätsverlusten, Arbeitsausfällen, Fehlzeiten und Arbeitslosigkeit, während sie auf ihre richtige Diagnose warten, da die Belastung sowohl physisch als auch psychisch vorhanden ist. Die dadurch entstehenden Kosten können die direkten Kosten deutlich übersteigen [13]. Diese indirekten Kosten, die durch Krankheit, Tod, Arbeitsausfall oder Verringerung der Arbeitsleistung entstehen, quantifizieren den gesamtwirtschaftlichen Produktivitätsverlust.

## Fallbeispiel

Am Beispiel eines Patienten, der besonders lange auf die richtige Diagnose warten musste, sollen die Möglichkeiten digitaler Diagnoseunterstützungssysteme skizziert werden (in diesem Fall des Systems Ada DX). Zum besseren Verständnis wird die Krankheitsgeschichte des Patienten (43 Jahre alt) kurz und bündig dargelegt:

- Seit dem vierten Lebensjahr abdominelle Schmerzen und Fieberschübe
- In der Folge rezidivierend auftretende Konjunktivitis und periorbitales Ödem
- Nachweis von erhöhter Blutkörperchensenkungsgeschwindigkeit, erhöhtem C‑reaktivem Protein und Leukozytose
- Im zwölften Lebensjahr Erstdiagnose einer Mesenteritis. Damals Probelaparotomie
- Im 25. Lebensjahr Perikarditis und Epididymitis
- Seit 2013 Proteinurie, 2015 3,4 g/g Kreatinin
- Nierenbiopsie 4/2015: AA-Amyloidose in der Niere
  → Diagnose Tumornekrosefaktor-Rezeptor-assoziiertes periodisches Syndrom (TRAPS) nach 39 Jahren

**Figure 2:**
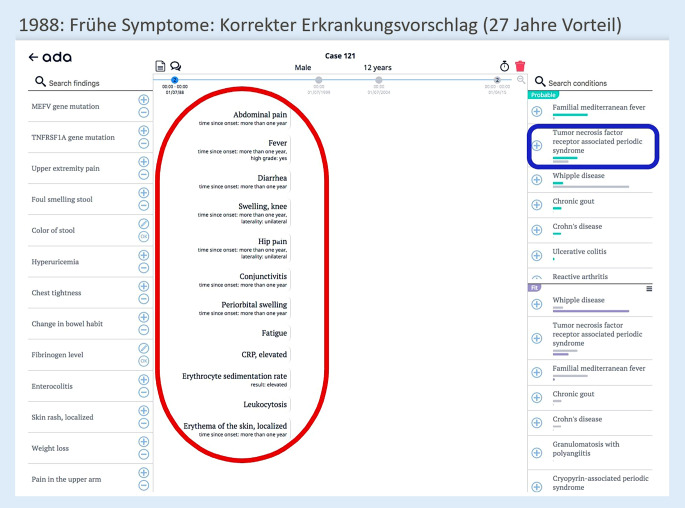

Der Patient wurde 1988 zum ersten Mal bei einer Ärzt:in vorstellig. Die damals dokumentierten Symptome führen bereits dazu, dass Ada DX im Jahr 1988 unter anderem die richtige Diagnose vorgeschlagen hätte (Abb. 2; [18]).

Insgesamt 10 weitere dokumentierte Untersuchungen wurden in den Jahren 2003 und 2004 vorgenommen, ohne erfolgreiche Diagnose. Im Jahr 2015 wurde der Patient an eine Expert:in überwiesen. Bereits 10 Tage später konnte die richtige Diagnose von der Expert:in für seltene Erkrankungen gestellt werden. Die direkten Kosten (6043,17 €) hätten für diesen Patienten eingespart werden können. Wesentlich bedeutender sind allerdings die Beeinträchtigungen der Lebensqualität des Patienten und die indirekten Kosten, die 27 Jahre lang akkumulierten.

**Figure 3:**
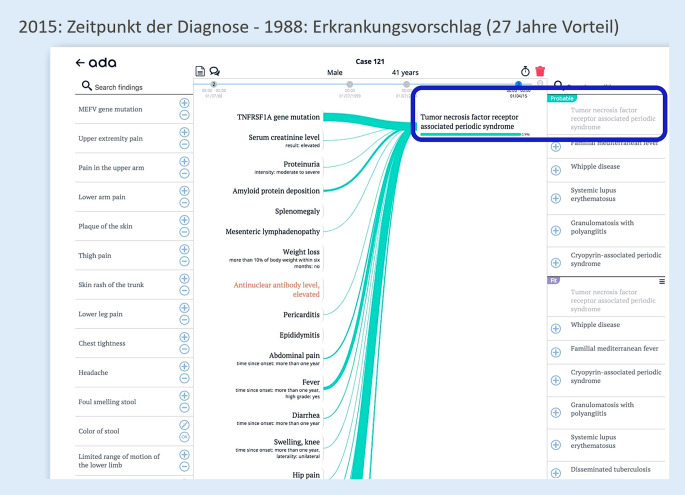

An diesem Fallbeispiel zeigt sich, wie digitale Diagnoseunterstützungssysteme Ärzt:innen und Expert:innen dabei helfen können, schneller eine richtige Diagnose bei Patient:innen mit seltenen Erkrankungen zu stellen. Zukünftig könnte sich sogar etablieren, dass Patient:innen mithilfe eines digitalen Diagnoseunterstützungssystems selbstständig ihre Symptome eingeben und als Ergebnis ein Tableau mit begründeten Diagnosevorschlägen erhalten. Dieses können sie bei ihrem darauffolgenden Arztbesuch vorlegen und den Diagnoseprozess somit optimieren [9]. Neben den Einsparungspotenzialen im Gesundheitssystem und einer Reduzierung der volkswirtschaftlichen Kosten könnten davon insbesondere die Patient:innen hinsichtlich ihrer Lebensqualität profitieren.

## Besondere Anforderungen an die künstliche Intelligenz

Training und Testung heutiger KI-Systeme erfordern viele hochwertige Datensätze. Damit ein solches KI-System eine seltene Erkrankung als solche erkennen kann, braucht es mehrere Hundert oder besser Tausende von hochwertigen Fallbeschreibungen. Genau diese gibt es aber für seltene Erkrankungen nicht, da sie eben selten sind. Daher finden sich im Bereich der seltenen Erkrankungen auch eher probabilistische und hybride KI-Systeme, die nicht auf konventionelles maschinelles Lernen setzen, sondern Wahrscheinlichkeitsmodelle verwenden oder solche Modelle mit neuronalen Netzen verbinden. Insgesamt wäre es aus Sicht der seltenen Erkrankungen erfreulich, wenn wir neuartige Algorithmen des maschinellen Lernens entwickeln würden, die mit erheblich weniger Datensätzen zu deutlich besseren Kategorisierungsergebnissen kämen. Hier ist zu wünschen, dass sich Forschungsförderprogramme der Entwicklung derartiger Algorithmen annehmen und so auch der Entwicklung von KI-Systemen im Bereich der seltenen Erkrankungen Vorschub leisten.

Aber nicht nur in der Diagnoseunterstützung kann KI helfen, sondern auch in der Therapiefindung und bei der Entwicklung geeigneter Medikamente für seltene Erkrankungen. Dabei werden neuerdings Transformer-basierte Verfahren zum Codieren und Textverstehen so miteinander kombiniert, dass aus großen Textmengen Beziehungen zwischen biomedizinischen Entitäten extrahiert und statistisch ausgewertet werden können [26]. Mithilfe solcher Verfahren werden dann biomedizinische Literaturdatenbanken und dort beschriebene Relationen mit verfügbaren Datenbanken und weiteren biochemischen Informationen abgeglichen, um einen Wissensgraphen zu bauen, der Menschen oder weiteren KI-Algorithmen Hinweise auf relevante Zusammenhänge zwischen pharmakologischen Zielen und aussichtsreichen Wirkstoffen geben kann. In diese Kategorie fallen auch Methoden des „re-purposing“ oder „re-positioning“ bekannter Wirkstoffe, die ebenfalls für die Therapiefindung bei seltenen Erkrankungen an Wichtigkeit gewinnen [27].

## Ethik

Bei den seltenen Erkrankungen liegen Chancen und Risiken des Einsatzes von KI sehr nahe beieinander. Daten- und Rechtssicherheit sowie Funktionalität sind kritische Faktoren im Kontext von Diagnosestellung und Therapieplanung. Insbesondere hinsichtlich der Rechtssicherheit gibt es Handlungsbedarf, um Ärzt:innen einen rechtssicheren Umgang mit digitalen Lösungen zu bieten. Wenn im Kontext seltener Erkrankungen digitale Unterstützungssysteme eine Diagnoseempfehlung abgeben, dient dies Ärzt:innen als Hilfsmittel zur schnelleren Diagnosestellung. Entscheidungshoheit und Verantwortung liegen also weiterhin vollständig bei der Ärzt:in. In einer solchen Situation untergräbt eine intransparente KI (Blackbox) die notwendige Transparenz der ärztlichen Entscheidungsfindung und erschwert es der Patient:in, diese nachzuvollziehen [25]. Wichtige Aspekte sind dementsprechend die Nachvollziehbarkeit der KI-Empfehlung, die Sicherheit der eingepflegten Patientendaten und eine Gewährleistung, dass das digitale System sowohl von Erfolg als auch von Misserfolg lernt. Außerdem sind das Vertrauensverhältnis zwischen Ärzt:in und KI-Entwickler:in bzw. zwischen Ärzt:in und Patient:in wie auch das Vertrauen in die Technologie selbst von essenzieller Bedeutung [8].

## Fazit für die Praxis

- Die erfolgreiche Diagnose von seltenen Erkrankungen dauert häufig sehr lange und ist für Patient:innen mit viel Stress und Unsicherheiten verbunden.
- Digitale Diagnoseunterstützungssysteme können dabei helfen, die Diagnosestellung schneller zu bewerkstelligen. Auf Basis eingetragener Symptome unterbreiten sie einem Experten differenzierte Diagnosevorschläge, wodurch dieser gezielter und schneller eine Diagnose stellen kann.
- So könnten Kosten für das Gesundheitssystem verhindert und die Lebensqualität von Patient:innen erhöht werden.
- Neben der Gewährleistung der Datensicherheit muss auch die Rechtssicherheit für die behandelnden Ärzt:innen bei Verwendung unterstützender Systeme abgedeckt sein, um Klarheit bei Haftungsfragen zu schaffen.
